# MST1 controls murine neutrophil homeostasis *via* the G-CSFR/STAT3 axis

**DOI:** 10.3389/fimmu.2022.1038936

**Published:** 2022-12-23

**Authors:** Sergi Masgrau-Alsina, Lou Martha Wackerbarth, Dae-sik Lim, Markus Sperandio

**Affiliations:** ^1^ Institute of Cardiovascular Physiology and Pathophysiology, Walter Brendel Center of Experimental Medicine, Ludwig-Maximilians University Munich, Munich, Germany; ^2^ Department of Biological Sciences, Korea Advanced Institute of Science and Technology (KAIST), Daejeon, Republic of Korea

**Keywords:** MST1, neutrophils, G-CSF, STAT3, granulocytes

## Abstract

The release of neutrophils from the bone marrow into the blood circulation is essential for neutrophil homeostasis and the protection of the organism from invading microorganisms. Granulocyte colony-stimulating factor (G-CSF) plays a pivotal role in this process and guides granulopoiesis as well as the release of bone marrow neutrophils into the blood stream both during homeostasis and in case of infection through activation of the G-CSF receptor/signal transduction and activation of transcription 3 (STAT3) signaling pathway. Here, we investigated the role of the mammalian sterile 20-like kinase 1 (MST1) for neutrophil homeostasis and neutrophil mobilization. We found increased plasma levels of G-CSF in *Mst1* ^-/-^ mice compared to wild type mice both under homeostatic conditions as well as after stimulation with the proinflammatory cytokine TNF-α. In addition, G-CSF-induced mobilization of neutrophils from the bone marrow into the blood circulation *in vivo* was markedly reduced in the absence of MST1. Interestingly, this was not accompanied by differences in the number of blood neutrophils. Addressing the underlying molecular mechanism of MST1-regulated neutrophil mobilization, we found reduced STAT3 phosphorylation and impaired upregulation of CXCR2 in *Mst1* ^-/-^ bone marrow neutrophils compared to wild type cells, while JAK2 phosphorylation was not altered. Taken together, we identify MST1 as a critical modulator of neutrophil homeostasis and neutrophil mobilization from the bone marrow, which adds another important aspect to the complex role of MST1 in regulating innate immunity.

## 1 Introduction

In humans, neutrophils are the most abundant leukocyte in the blood circulation. They have a rather short life span of 10 – 12 hours (1) before they either get activated and recruited into inflamed tissue or become senescent and are cleared from the circulation by the spleen, bone marrow or lung (2). Consequently, mechanisms controlling neutrophil homeostasis ranging from neutrophil generation in the bone marrow (granulopoiesis), its subsequent release into the bloodstream, and finally its clearance from the blood circulation need to function properly to ensure appropriate levels of circulating neutrophils to protect the organism.

In case of inflammation, the increased neutrophil demand requires emergency granulopoiesis and neutrophil mobilisation. Granulocyte-colony stimulating factor (G-CSF) has been identified as the principal cytokine regulating both processes (3). In humans, G-CSF serum levels are nearly undetectable under baseline conditions but rapidly increase during inflammation (4). Changes in G-CSF serum levels is under control of interleukin (IL) lL-23 and IL-17. Production and release of IL-23 from macrophages or dendritic cells induces neutrophil-regulatory T cells (Tn) to produce IL-17 that finally induces the release of G-CSF by endothelial cells, fibroblasts, bone marrow stroma cells and several immune cells (5, 6). Upon binding of G-CSF to the G-CSF receptor (G-CSFR), G-CSFR homodimerizes and activates a signaling transduction cascade leading to the induction of enhanced granulopoiesis and neutrophil mobilization into the blood stream. The JAK2-STAT3 axis is the principal signaling pathway activated upon G-CSFR stimulation in granulocyte-monocyte progenitors (GMPs) and downstream neutrophil precursor cells in the bone marrow (7). Hereby, STAT3 acts as a double-edged sword in granulopoiesis. On the one hand, STAT3 promotes neutrophil differentiation by inducing the expression of members of the C/EBP family of transcription factors (8). On the other hand, STAT3 has a negative role in emergency granulopoiesis through the promotion of SOCS3 expression, a STAT-induced STAT inhibitor (SSI) that suppresses G-CSF induced signaling (9). STAT3 has also a leading role in G-CSF-dependent neutrophil mobilization by controlling expression of chemokine receptors CXCR4 and CXCR2. While CXCR4 confers a strong retaining signal by binding to CXCL-12 (also known as SDF-1), CXCR2 engagement by CXCL8 in humans (and CXCL1 in mice) has the opposite effect promoting neutrophil mobilization into the blood circulation. Accordingly, activated STAT3 induces the release of neutrophil proteases that break CXCR4/CXCL12 bonds (10) and at the same time promotes the upregulation of CXCR2 expression on the neutrophil surface through Raf/MEK/ERK dependent signaling pathways (11).

Once in the blood circulation, neutrophils get recruited to sites of inflammation in a multistep process that requires the sequential action of a whole array of adhesion relevant molecules (12, 13). Recently, mammalian sterile 20-like kinase 1 (MST1, also known as serine/threonine kinase 4, STK4) has been reported to play an essential role in penetration of the vascular basement membrane, the last step along the neutrophil recruitment cascade, by controlling VLA-3, VLA-6, and neutrophil elastase-containing vesicle mobilization to the neutrophil surface (14). MST1 is widely known to be the central kinase of the mammalian Hippo pathway, first described as a major regulator of organ size during development (15) and later also found to be involved in the control of cell proliferation, apoptosis (16, 17), and differentiation (18). Through phosphorylation of substrates other than those from its canonical pathway, MST1 also emerged as important modulator of immune cell function such as leukocyte adhesion (19) or the aforementioned neutrophil vascular basement membrane penetration (14). Here, we report a novel function of MST1 in neutrophil homeostasis affecting neutrophil mobilization and release from the bone marrow to the circulation, triggered by a regulatory role of MST1 in G-CSF/G-CSFR-dependent activation of the JAK2/STAT3 signaling pathway.

## 2 Methods

### 2.1 Animal models

All experiments were performed in mice with a C57BL/6 background. The knockin strain *Lyz2^eEGP^* (neutrophil reporter strain) and the knockout strain *Mst1^-/-^* were generously provided by Dr. Thomas Graf (Center for Genomic Regulation, Barcelona, ES) and Dr. Dae Sik Lim (KAIST, Daejeon, KR), respectively (20, 21). *Mst1^-/-^Lyz2^eEGP^* mice were generated by crossing the two aforementioned mouse lines. All animals were bred in the animal facility of the Biomedical Centre of Munich (BMC) and experiments were conducted under the approval of the Regierung von Oberbayern (ROB) and local authorities (project number ROB-55.2-2532.Vet_02-17-102).

### 2.2 Neutrophil release assay

C57BL/6 and *Mst1^-/-^* mice were anesthetised with a combined intraperitoneal injection of 125mg·kg^-1^ ketamine (Zoetis) and 12,5mg/kg^-1^ xylazin (Bayer) for blood collection and intravenous injection of NaCl 0.9% (control) or 2.5μg of recombinant murine G-CSF (ImmunoTools). Four hours after injection of NaCl 0.9% or G-CSF, mice were bled again and sacrificed. Blood was further processed and analyzed by flow cytometry.

### 2.3 Multi-photon imaging

*Lyz2^eEGP^* and *Mst1^-/-^Lyz2^eEGP^* mice were first stimulated with an intravenous injection of 2.5μg of recombinant murine G-CSF to induce neutrophil mobilisation from the bone marrow into the blood circulation (22). Shortly before imaging, mice were anaesthetised with 125mg·kg^-1^ ketamine (Zoetis) and 12,5mg/kg^-1^ xylazin. The skull bone was then exposed by removing the hair and the skin over it, as well as the pericranium with the gentle help of a scalpel. A metal ring was glued on the skull bone and firmly fixed to the imaging stage in order to prevent any breathing movement. To visualize the bone marrow microvasculature, 10μL of QTracker™ 705 (Thermo Fisher Scientific) was administrated intravenously. Imaging was carried out with the TCS SP8 MP Multiphoton Microscope (Leica) using the objective HC IRAPO L (25x, NA1.00 W, Leica) and started 2 hours after G-CSF injection. For each mouse, an area of analysis of 70x170μm was defined surrounding a vessel. Imaging was acquired in 512x512 pixels with a step size of 4μm for intervals of 15s from hour 2 to hour 4 post G-CSF injection. Analysis was performed using Imaris 8 Cell Imaging Software (Oxford Instruments).

### 2.4 Supplemental methods

Further experiments including bromodeoxyuridine cell proliferation assay, TNF-α induced peritonitis and serum G-CSF detection, as well as general procedures for flow cytometry and western blot can be found in **Supplemental Methods**.

### 2.5 Statistics

All statistical analyses and graphs were generated with GraphPad 9 (Prism) software. Statistical tests were performed according to the number of groups being compared. For pairwise comparison of experimental groups, an unpaired student’s t-test was performed. For more than two groups, a one-way or two-way analysis of variance (ANOVA) with either Tukey’s (one-way ANOVA) or Sidak’s (two-way ANOVA) post-hoc test was used, respectively. P-values <0.05 were considered statistically significant.

### 2.6 Data availability statement

For original data, please contact markus.sperandio@lmu.de.

## 3 Results

### 3.1 *Mst1* ^-/-^ mice show increased numbers and proliferation of neutrophils in the bone marrow

**Figure 1:**
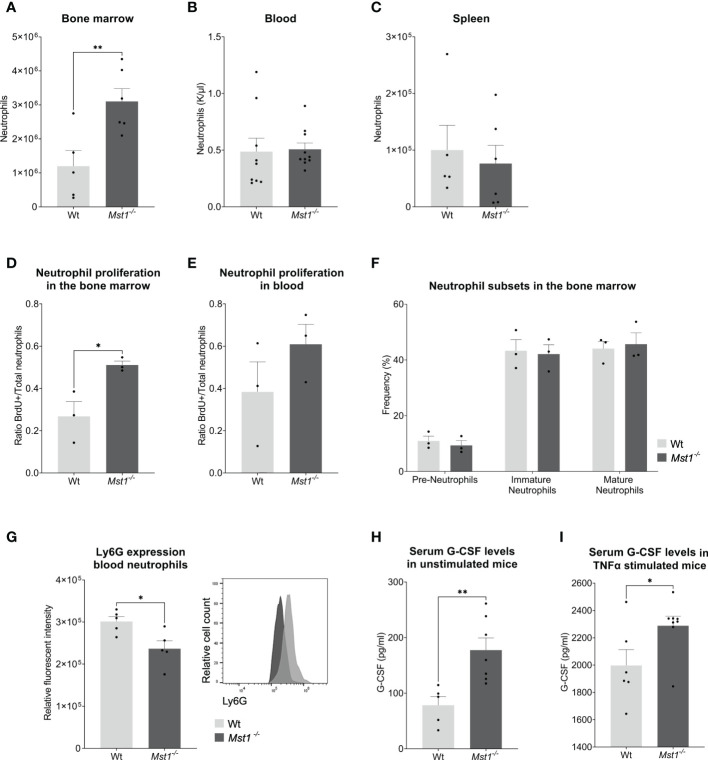
*Mst1^-/-^* mice show increased numbers of neutrophils in the bone marrow and higher levels of G-CSF under normal conditions and after TNF-α stimulation. **(A–C)** Neutrophil counts measured from blood with a haematology analyser **(B)** or from two femurs and whole spleen with flow cytometry **(A, C)**; **(A, C)** n=5/6, **(B)** n=9/10, **(D, E)** Ratio of BrdU^+^ neutrophils per total number of neutrophils (Ly6G^+^) in bone marrow **(D)** and peripheral blood **(E)** 2 days after BrdU/G-CSF i.v. injection; n=3. **(F)** Frequencies of neutrophil subsets in the bone marrow obtained by flow cytometry; n=3. **(G)** Expression of Ly6G on peripheral blood neutrophils; n=5. **(H, I)** Serum G-CSF levels from unstimulated and stimulated mice measured by ELISA. **(H)** n=5/7, **(I)** n=6/8. Unpaired t-test; *p<0.05, **p<0.01.

First, we analysed absolute numbers of bone marrow cells by flow cytometry and found that bone marrow resident neutrophils were significantly increased in *Mst1^-/-^* mice compared to wild type (WT) mice (**Figure 1A**). Next, we assessed neutrophil numbers in the peripheral blood. Here, we could not detect any difference in the number of circulating neutrophils between WT and *Mst1^-/-^* mice (**Figure 1B**). After 10-12 hours in circulation, neutrophils enter senescence and migrate mostly towards the spleen to get cleared from the circulation. Comparable to the blood compartment, we found similar numbers of neutrophils in the spleen of WT and *Mst1^-/-^* mice (**Figure 1C**) suggesting that neutrophil clearance in the periphery is not significantly affected in *Mst1* ^-/-^ mice.

The lack of MST1 has been previously reported to cause higher numbers of hematopoietic stem and progenitor cells (HSPCs) in the bone marrow due to increased proliferation (17). In order to determine whether this was also the case for bone marrow-resident neutrophils, we injected mice intravenously with 2.5mg of BrdU followed by 2.5µg of G-CSF one hour later to stimulate neutrophil differentiation and release (23). Two days later, we analyzed the number of newly generated neutrophils in the bone marrow and blood using flow cytometry. We observed a higher ratio of BrdU^+^ neutrophils in *Mst1^-/-^* bone marrow (**Figure 1D**) suggesting that (similar to HSPCs) bone marrow-resident neutrophils were also more proliferative. This tendency could not be observed in circulating neutrophils (**Figure 1E**). After having shown an increase in number and proliferative capacity of neutrophils in the bone marrow, we became interested whether this observation is due to defective maturation of neutrophils from the granulocyte-macrophage progenitor (GMP) state to mature neutrophils. Therefore, we assessed the various neutrophil precursor subsets as recently described by Evrard and colleagues (24). Interestingly, neutrophil subset frequencies were similar in *Mst1^-/-^* compared to WT mice (**Figure 1F**) ruling out a maturation stop during neutrophil differentiation in the bone marrow. After bone marrow neutrophils get released to the circulation they experience a process of aging that affects their function (25). Recently, it was shown that an increase in Ly6G expression correlates with neutrophil aging (26). We found decreased expression of Ly6G on circulating *Mst1^-/-^* neutrophils (**Figure 1G**), suggesting a reduction of aged neutrophils in the blood circulation of mice lacking MST1.

### 3.2 *Mst1* ^-/-^ mice have increased levels of plasma G-CSF under normal conditions and during inflammation

Our observation of an increase in neutrophils and its precursors in the bone marrow of *Mst1^-/-^* mice did not translate into higher numbers of circulating neutrophils. One potential explanation for this might be that neutrophil mobilization and release into the blood circulation are impaired in the absence of MST1. To address this, we first assessed serum G-CSF levels in the blood circulation. G-CSF is not only the most important factor for neutrophil differentiation but also critical for the mobilization and release of neutrophils into the circulation (27). Serum G-CSF levels were investigated in unstimulated mice and in mice stimulated with intraperitoneal injection of TNF-α. We found that *Mst1^-/-^* mice had higher baseline serum levels of G-CSF compared to WT mice (**Figure 1H**). In addition, we observed an increase in serum G-CSF levels 4 hours after intraperitoneal injection of TNF-α which was more pronounced in *Mst1* ^-/-^ mice compared to WT mice (**Figure 1I**) implying defective G-CSF signaling in neutrophils and its precursors in the absence of MST1.

### 3.3 G-CSF-induced mobilization of bone marrow neutrophils into the circulation is impaired in the absence of MST1

**Figure 2:**
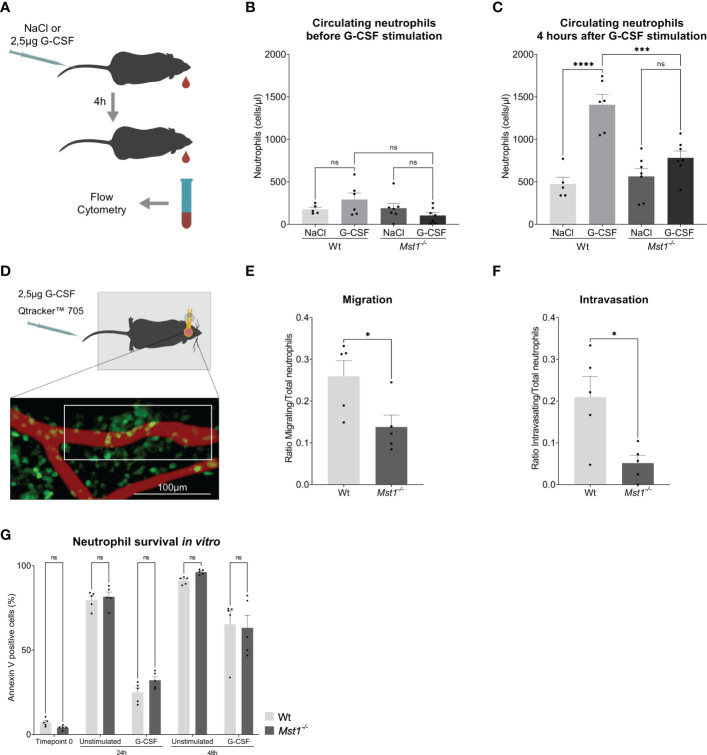
*Mst1* ^-/-^ neutrophils show an impaired response to G-CSF stimulation. **(A)** Schematic representation of the neutrophil release assay. **(B, C)** Flow cytometry analysis of blood circulating neutrophils before and after G-CSF stimulation; n=5-7. **(D)** Schematic representation of the two-photon skull imaging. The highlighted area of analysis is 70x170μm. **(E, F)** Number of cells migrating **(E)** and intravasating the vessel **(F)** relative to the total number of cells at T_0_ in the area of analysis; n=5. **(G)** Flow cytometry analysis of Annexin V staining in unstimulated and G-CSF stimulated isolated neutrophils; n=5. One-way ANOVA; ns, non significant; ***p<0.001, ****p<0.0001 **(B, C)**, unpaired t-test; *p<0.05 **(E, F)** and two-way ANOVA **(G)**.

In order to confirm the defective response of *Mst1* ^-/-^ neutrophils to G-CSF, we injected 2.5µg of G-CSF intravenously and counted peripheral blood neutrophils 4 hours after injection (**Figure 2A**). While G-CSF-injection provoked a significant increase of circulating neutrophils in WT mice, we found no significant increase in *Mst1^-/-^* mice (**Figures 2B, C**). Using intravital multiphoton laser scanning microscopy and a murine skull bone marrow window model (**Figure 2D**), we next investigated the defective G-CSF-induced mobilization of neutrophils from the bone marrow into the circulation in the absence of MST1. To visualize neutrophils in *Mst1* ^-/-^ mice, we crossed *Lyz2* ^eGFP^ mice with *Mst1* ^-/-^ mice (*Mst1^-/-^Lyz2^eEGP^* mice) and used *Lyz2* ^eGFP^ as controls. While no mobilisation could be perceived in unstimulated mice (Video 1), we observed GFP^+^ cell mobilization within the bone marrow and intravasation 2 to 4 hours after G-CSF injection. During that time we assessed how many GFP^+^ cells showed a migratory behaviour within the bone marrow and quantified the numbers of GFP^+^ cells intravasated into the microvasculature. Interestingly, the number of migratory GFP^+^ cells within the bone marrow was significantly decreased in *Mst1^-/-^Lyz2^eEGP^* mice compared to *Lyz2^eEGP^* mice (**Figure 2E**, Videos 2 and 3). In addition, we found significantly reduced numbers of intravasating neutrophils in *Mst1^-/-^Lyz2^eEGP^* mice compared to *Lyz2^eEGP^* mice (**Figure 2F**) suggesting that neutrophil trafficking from the bone marrow into the circulation is impaired in the absence of MST1.

Finally, we were testing whether regulation of granulopoiesis of bone marrow isolated Ly6G positive cells stimulated with G-CSF for 48h was different on the level of apoptosis induction between WT and *Mst1^-/-^* mice. G-CSF had been reported to prolong neutrophil survival by blocking apoptosis (28). Accordingly, we analyzed Annexin V staining of untreated and G-CSF-treated bone marrow derived neutrophils and observed the described protective role of G-CSF on neutrophil survival, but no differences were found in the rate of apoptosis between WT and *Mst1^-/-^* mice for all timepoints tested (**Figure 2G**). This suggests that MST1 does not regulate apoptosis in unstimulated or G-CSF-stimulated bone marrow derived neutrophils.

### 3.4 G-CSF-induced JAK2/STAT3 signaling axis is impaired in *Mst1^-/-^* mice

**Figure 3:**
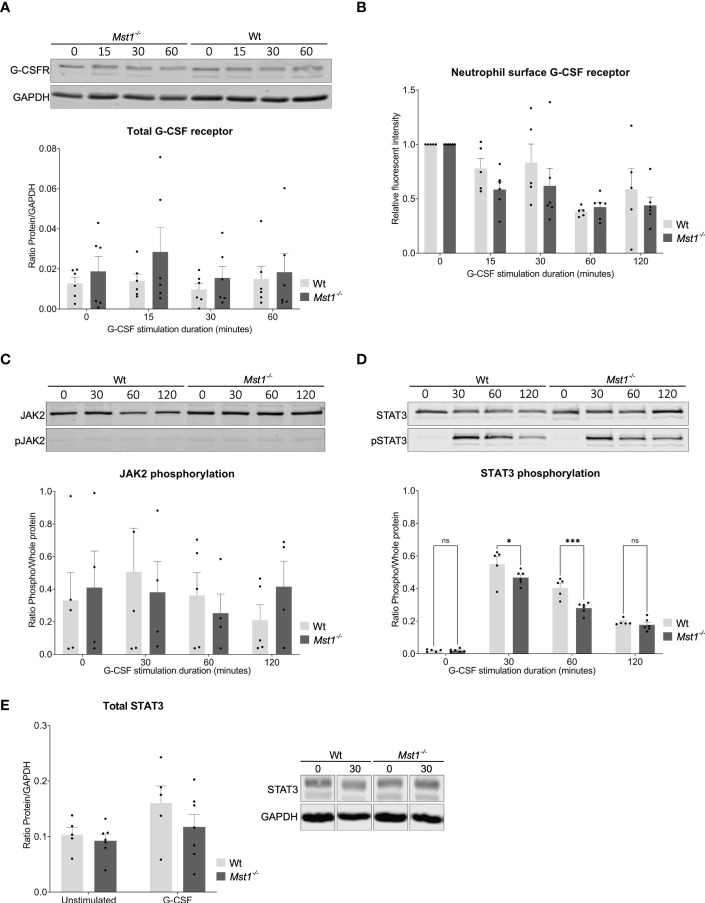
STAT3 phosphorylation reduced in *Mst1* ^-/-^ neutrophils after G-CSF stimulation. **(A)** Western blot analysis of total G-CSFR levels in unstimulated and G-CSF stimulated purified bone marrow neutrophils normalised to GAPDH; n=6. **(B)** Fold change of membrane levels of G-CSFR in G-CSF stimulated relative to unstimulated bone marrow neutrophils; n=5. **(C)** Neutrophil JAK2 phosphorylation normalized to total JAK2; n=5. **(D)** Neutrophil STAT3 phosphorylation normalized to total STAT3; n=5. **(E)** Total levels of STAT3 analysed by western blot in unstimulated and 30 minute G-CSF stimulated purified bone marrow neutrophils from WT and *Mst1^-/-^* mice; n=5. Two-way ANOVA; ns, non significant; *p<0.05, ***p<0.001.

To elucidate the molecular mechanisms responsible for defective neutrophil mobilization of bone marrow neutrophils into the circulation in the absence of MST1, we investigated G-CSFR dependent signaling events in neutrophils from *Mst1^-/-^* and WT mice. We first assessed overall G-CSFR levels in isolated bone marrow neutrophils by western blot. We found no difference in total G-CSFR expression in neutrophils that were either unstimulated or stimulated with G-CSF for 15, 30 and 60 minutes (**Figure 3A**). Upon binding of G-CSF to G-CSFR, G-CSFR is internalized and can be recycled in a process that highly depends on vesicular trafficking (29). Since MST1 was previously reported to regulate vesicle trafficking (14, 30), we hypothesized that the recycling route could be altered and plasma membrane levels of G-CSFR were altered after exposure to G-CSF. Therefore we investigated membrane levels of G-CSFR in neutrophils that were exposed to G-CSF for 15, 30, 60 and 120 minutes using flow cytometry. We did not find a significant difference in the levels of surface G-CSFR for any of the time points investigated (**Figure 3B**), excluding different G-CSFR surface expression levels between *Mst1^-/-^* and WT neutrophils to account for the impaired mobilization of bone marrow neutrophils into the circulation in the absence of MST1.

Engagement of G-CSFR activates several signaling pathways that depend on phosphorylation of respective signaling molecules. We screened for phosphorylation patterns of several proteins downstream of G-CSFR after neutrophils were stimulated with G-CSF. We did not find any differences in JAK2, JAK1, STAT5, AKT, ERK, and MAPK phosphorylation (**Figure 3C**, data not shown). In contrast, we observed significantly diminished phosphorylation of STAT3 in *Mst1^-/-^* compared to WT neutrophils after 30 and 60 minutes of G-CSF stimulation (**Figure 3D**). To elucidate whether MST1 also regulates overall STAT3 expression, we assessed the levels of total STAT3 by western blot and found similar expression levels of total STAT3 in unstimulated and G-CSF-stimulated *Mst1^-/-^* and WT neutrophils (**Figure 3E**) suggesting that MST1 influences STAT3 phosphorylation but not overall STAT3 expression levels.

**Figure 4:**
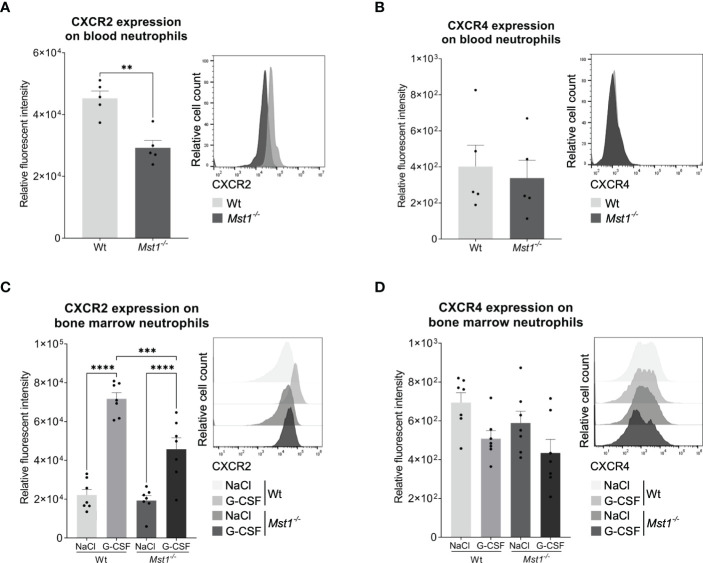
STAT3 downstream target CXCR2 is reduced in *Mst1* ^-/-^ neutrophils after G-CSF stimulation. **(A, B)** CXCR2 **(A)** and CXCR4 **(B)** expression in peripheral blood neutrophils of unstimulated mice; n=5. **(C, D)** CXCR2 **(C)** and CXCR4 **(D)** expression in bone marrow neutrophils 1 day after *in vivo* G-CSF stimulation; n=7. Unpaired t-test; **p<0.05 **(A, B)**. Two-way ANOVA **(C, D)**; ***p<0.001, ****p<0.0001.

Activated STAT3 acts as a transcription factor promoting the expression of genes enhancing neutrophil mobilization such as chemokine receptor CXCR2 (11). Therefore, we first evaluated the levels of CXCR2 and CXCR4 on the surface of circulating neutrophils under baseline conditions. Interestingly, CXCR2 expression was reduced in *Mst1^-/-^* neutrophils (**Figure 4A**), while CXCR4 surface levels were unaltered (**Figure 4B**). The expression of CXCR2 and CXCR4 on bone marrow neutrophils was similar between WT and *Mst1^-/-^* unstimulated mice (**Figures 4C, D**). However, 24h after G-CSF stimulation *Mst1^-/-^* bone marrow neutrophils failed to upregulate CXCR2 in contrast to WT neutrophils (**Figure 4C**) indicating that G-CSF-induced upregulation of CXCR2 surface expression in neutrophils is impaired in the absence of MST1.

## 4 Discussion

The serine/threonine kinase MST1 is a critical factor in regulating various biological processes including organ growth and morphology, cell proliferation and apoptosis through activation of the canonical Hippo signaling pathway (31). Recently, several reports have revealed that MST1 is also involved in various immune cell functions through MST1-dependent non-canonical signaling events (32, 33). Here, we expand this knowledge and demonstrate that MST1 regulates G-CSF receptor-dependent signaling affecting neutrophil homeostasis *in vivo.* Specifically, we show that *Mst1* ^-/-^ mice fail to mobilize neutrophils from the bone marrow into the blood circulation and that this impaired response is due to diminished activation of STAT3, one of the major transcription factors activated by G-CSF receptor signalling.

In humans, nonsense mutations that cause a functional deficiency of the protein MST1 provoke an immunodeficiency syndrome characterized by severe lymphopenia, transient neutropenia, and recurrent bacterial and viral infections (34, 35). Interestingly, *Mst1* ^-/-^ mice did not present any alterations in circulating blood neutrophil counts, but showed defective neutrophil transendothelial migration into inflamed tissue (14). Of note, loss of both MST1 and MST2 even leads to neutrophilia, as reported recently (17). This might reflect a more pronounced neutrophil trafficking defect in *Mst1/2* double knockout conditions than in *Mst1*-deficient mice leading to the inability of circulating neutrophils to extravasate into tissue. Remarkably, neutrophilia is also a hallmark of leukocyte adhesion deficiencies I-III (36). Interestingly, in contrast to the normal blood neutrophil counts observed in *Mst1* ^-/-^ mice, the absolute numbers of bone marrow resident neutrophils were significantly increased in *Mst1* ^-/-^ mice. This was associated with an enhanced generation of neutrophils in the bone marrow of *Mst1* ^-/-^ mice, a finding which is in agreement with an earlier study by Lee and colleagues in *Mst1/2* double deficient mice, where the authors described an increased pool of hematopoietic stem and precursor cells (HSPCs) and hypercellularity of the bone marrow (17).

Although we did not detect neutropenia in the absence of MST1, the fact that high bone marrow neutrophil numbers do not translate into higher counts in circulating neutrophils might point towards defective differentiation of myeloid precursor cells to mature neutrophils and/or an impaired release of bone marrow-derived neutrophils into the blood circulation. To investigate this, we analyzed neutrophil subset frequencies in the bone marrow, which revealed that *Mst1* ^-/-^ mice do not have a neutrophil differentiation defect. We then focused on the mobilization of neutrophils from the bone marrow into the circulation, which is controlled by G-CSF both under steady state and inflammatory conditions (29). The assessment of G-CSF serum levels revealed that both under resting conditions as well as following stimulation with the proinflammatory cytokine TNF-α, G-CSF levels were higher in *Mst1* ^-/-^ than WT mice suggesting that loss of MST1 leads to impaired G-CSF-dependent mobilization of neutrophils into the blood circulation. This could be confirmed in experiments where we injected G-CSF and assessed neutrophil counts 4h after G-CSF treatment. This led to a significant increase in blood neutrophil counts in WT but not in *Mst1* ^-/-^ mice. The role of G-CSF and G-CSFR in regulating blood neutrophil counts was clearly demonstrated in *Csf3* ^-/-^ (G-CSF deficient) and *Csf3r* ^-/-^ (G-CSFR deficient) mice in response to an infection (28, 37, 38).

To further explore why *Mst1* ^-/-^ neutrophils did not appear in the circulation upon G-CSF stimulation, we evaluated G-CSF induced neutrophil migration in *Mst1^-/-^Lyz2^eEGP^* and *Lyz^eGFP^* mice using two-photon laser scanning microscopy and observed reduced neutrophil motility within the bone marrow. In addition, we found a reduced number of intravasating neutrophils. Similar results had been obtained in *Csf3r* ^-/-^ mice (28, 37–39). Currently, it is unclear whether reduced intravasation of neutrophils from the bone marrow into the blood circulation in *Mst1* ^-/-^ mice is linked to the described defect in trans-endothelial migration of *Mst1^-/-^* neutrophils into inflamed tissue. This defect is caused by impaired intracellular mobilization of VLA-3, VLA-6, and NE-containing vesicles to the neutrophil surface (14, 19), a prerequisite for vascular basement membrane penetration. Of note, bone marrow sinusoidal vessels show a discontinuous endothelial lining affecting also the vascular basement membrane (40), which might facilitate the intravasation of neutrophils into the blood circulation.

To identify molecular mechanisms, which might explain how MST1 regulates granulopoiesis and neutrophil homeostasis, we investigated G-CSF/G-CSFR dependent signaling events. G-CSFR is a transmembrane protein that upon G-CSF binding activates several signalling pathways, such as PIK3/Akt, MAPK, and JAK-STATs (41, 42). The G-CSF induced activation leads to receptor internalization, that can then follow two pathways: degradation *via* the lysosome or recycling through the endoplasmic reticulum and Golgi apparatus (29). As MST1 was reported to interact with the vesicular trafficking machinery in immune cells (14, 19), we looked for alterations in G-CSFR expression. We did not observe any changes neither in the overall G-CSFR levels nor in the G-CSFR membrane levels during G-CSF stimulation indicating that receptor degradation and recycling are not altered in *Mst1* ^-/-^ mice. The major signalling cascade activated downstream of G-CSFR is the JAK2-STAT3 signaling pathway. Phosphorylation patterns of JAK2 and STAT3 in *in vitro* G-CSF-stimulated neutrophils revealed no difference for JAK2 between WT and *Mst1^-/-^* neutrophils. However, STAT3 phosphorylation was significantly diminished in the absence of MST1, although total STAT3 levels in both unstimulated and G-CSF-stimulated neutrophils were not affected in the absence of MST1. Interestingly, studies in *Stat3* deficient mice showed that STAT3 is dispensable for neutrophil differentiation but affects G-CSFR dependent neutrophil mobilization (43, 44), although STAT3 can also promote the transcription of suppressor of cytokine signalling 3 (SOCS3) that negatively regulates G-CSFR signalling (45). In mature neutrophils, G-CSFR-activated STAT3 is key for neutrophil release from the bone marrow to the circulation (11). Accordingly, *Stat3* deficient mice show a similar phenotype after G-CSF stimulation (46) to that we observed in *Mst1* ^-/-^ mice. Following G-CSF binding to G-CSFR, STAT3 activation controls neutrophil mobilization by [1] downregulating CXCR4 ligands and upregulating CXCR2 ligands, [2] controlling the amplitude of CXCR2 signalling, and [3] directly promoting the transcription of CXCR2 (11). In our study we found that after *in vivo* application of G-CSF bone marrow resident *Mst1* ^-/-^ neutrophils had reduced expression of CXCR2 compared to WT mice which strongly indicates impaired signaling through the G-CSFR/JAK2/STAT3 axis in *Mst1* ^-/-^ mice.

**Figure 5:**
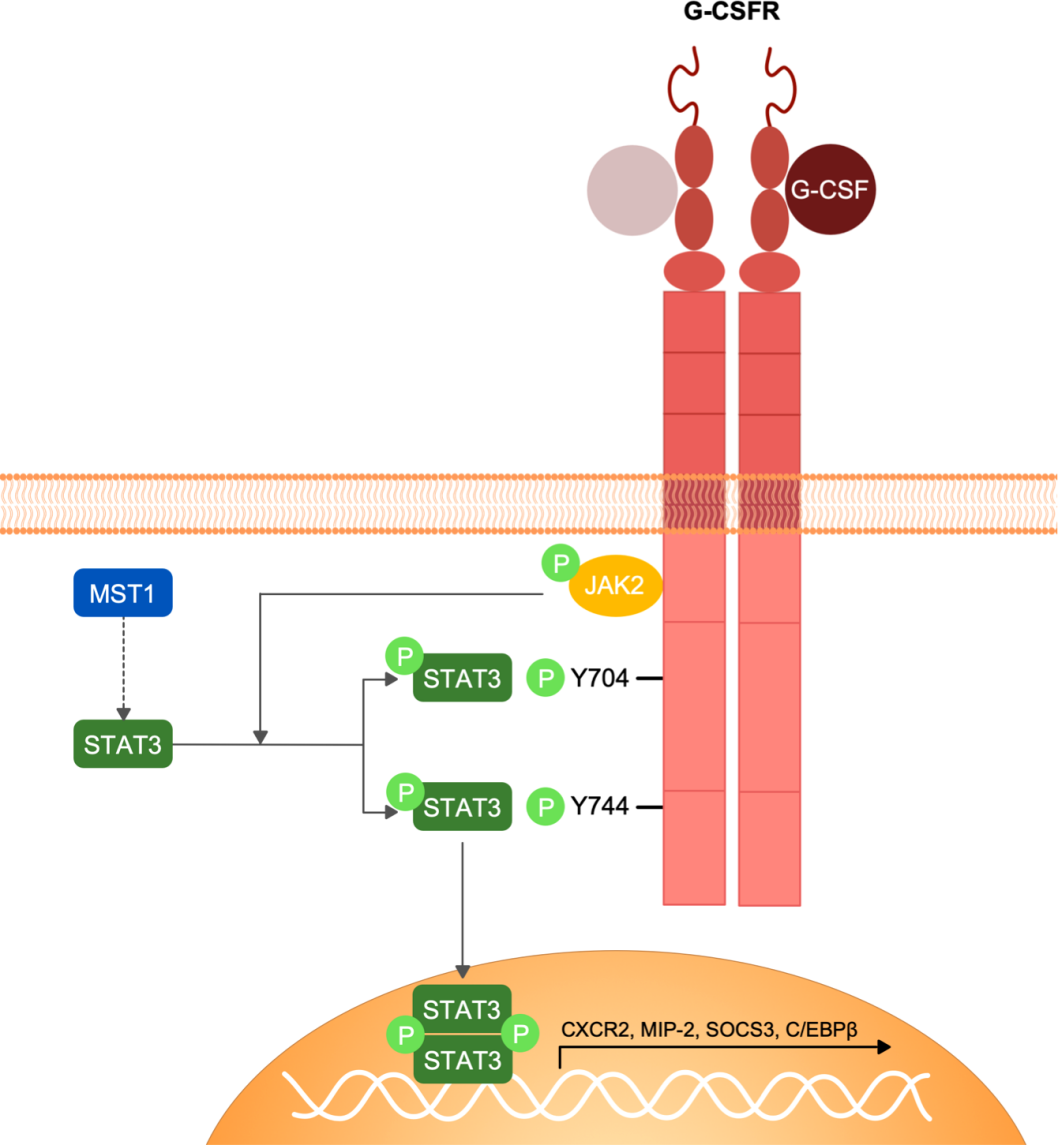
Schematic representation of G-CSFR signalling and MST1-dependent regulation of STAT3 phosphorylation. G-CSF interaction with G-CSFR induces the homo-dimerization of the receptor and the conformational change of its intracellular domain that favours the activation of JAK2 by trans-phosphorylation. JAK2 in turn phosphorylates G-CSFR and STAT3. STAT3 phosphorylation by JAK2 following G-CSFR stimulation is regulated by MST1 indicating an important role of MST1 in the G-CSF/JAK2-STAT3 signaling axis.

In conclusion, we have identified MST1 as an important regulator of the G-CSFR/STAT3 signalling pathway in neutrophils (**Figure 5**) that orchestrates neutrophil mobilization from the bone marrow into the blood circulation and hence neutrophil homeostasis. These insights expand the ever-growing non-canonical functions of MST1 in innate immune cells illustrating the complex role of this kinase in modulating the innate immune system and opens new therapeutic perspectives for those suffering from the MST1 absence-derived immunodeficiency.

## Data availability statement

The raw data supporting the conclusions of this article will be made available by the authors, without undue reservation.

## Ethics statement

The animal study was reviewed and approved by Regierung von Oberbayern project number ROB-55.2-2532.Vet_02-17-102.

## Author contributions

SM-A designed and conducted experiments, analyzed data and wrote the manuscript. LMW performed the neutrophil survival experiment. D-SL provided critical tools and edited the manuscript. MS set up the project, designed experiments and wrote the manuscript. All authors contributed to the article and approved the submitted version.
