# Supplementary material for: MST1 controls murine neutrophil homeostasis via the G-CSFR/STAT3 axis
Source: Front Immunol. 2022 Dec 23;13:1038936. doi: 10.3389/fimmu.2022.1038936 (PMC9816424; doi:10.3389/fimmu.2022.1038936)
Supplement: Supplementary file 1 [file DataSheet_1.pdf]

MST1 controls murine neutrophil homeostasis via the G-CSFR/STAT3 axis

Sergi Masgrau-Alsina[1], Lou Martha Wackerbarth[1], Dae-sik Lim[2], and Markus Sperandio[1]

[1]Institute of Cardiovascular Physiology and Pathophysiology, Walter Brendel Center of Experimental Medicine, Ludwig-Maximilians University Munich, Planegg-Martinsried, Germany

[2]Department of Biological Sciences, Korea Advanced Institute of Science and Technology (KAIST), Daejeon, South Korea

ORCIDs:
M.S.: 0000-0002-7689-3613

***Correspondence:**

Markus Sperandio, M.D.
Institute of Cardiovascular Physiology and Pathophysiology
Walter Brendel Center of Experimental Medicine
Biomedizinisches Centrum
Ludwig-Maximilians-Universität
Großhaderner Str. 9
82152 Planegg-Martinsried
GERMANY
Voice: +49 (0)89 2180 71513
Fax: +49 (0)89 2180 71511
Email: markus.sperandio@lmu.de

Key words: MST1, neutrophils, G-CSF, STAT3, granulocytes.

Running title: MST1 and neutrophil homeostasis

Supplemental content: supplemental methods, figures, references, tables, and videos.

Supplemental reference count: 2

Supplemental figures: 1

Supplemental tables: 1

Videos: 3

Supplemental Methods

Flow Cytometry

Isolated cells were stained for 30 minutes on ice in a solution of PBS-1%BSA or HBSS (250mg BSA, Capricorn Scientific; 238mg HEPES, Sigma-Aldrich,; 100mL Hank's solution, pH7.4) containing the manufacturer's recommended antibody concentration. Samples were analysed using Gallios (Beckman Coulter), CytoFlex S (Beckman Coulter), or Fortessa (BD Biosciences). Neutrophils were identified in bone marrow and spleen samples as $CD45^{+}$, $CD11b^{+}$, and $Ly6G^{+}$ cells and in blood samples as $CD45^{+}$, $CD11b^{+}$, $CD115^{-}$, and $Gr1^{high}$ cells. For identifying the neutrophil subsets in the bone marrow, the gating strategy described by Evrard and colleagues was applied (Evrard et al., 2018) (Supplemental Figure 1). For the neutrophil survival experiment, isolated neutrophils were incubated in HBSS with and without $1ug \cdot mL^{-1}$ G-CSF at 37ºC for 24 or 48h and later stained with FITC Annexin V Apoptosis Detection Kit with PI (BioLegend) following the manufacturer instructions. The complete list of the antibodies used can be found in Supplemental Table 1.

Bromodeoxyuridine (BrdU) cell proliferation assay

Bone marrow neutrophil proliferation in C57BL/6 and $Mst1^{-/-}$ mice was assessed using the APC BrdU Flow Kit (BD Biosciences) following the manufacturer's protocol. One hour after 2.5µg BrdU intravenous injection, 2.5µg recombinant murine G-CSF (ImmunoTools) was administrated intravenously to promote neutrophil proliferation. Mice were sacrificed and bone marrow isolated two days after the injection and further processed to be analysed by flow cytometry.

TNF-α induced peritonitis and G-CSF serum levels

C57BL/6 and $Mst1^{-/-}$ mice were injected with either NaCl (control) or 500ng recombinant murine TNF-α (R&D Systems) intraperitoneally to induce peritonitis, as described (Kurz et al., 2016). Serum was collected 4 hours after injection and processed as indicated by the protocol of Mouse G-CSF Quantikine® ELISA (R&D Systems) and read with the Spark™ 10M microplate reader (Tecan).

Western Blot

Purified bone marrow neutrophils were incubated in a solution of $1ug \cdot mL^{-1}$ G-CSF at 37ºC for different time points. Neutrophils were then immediately lysed and

homogenized in a lysis buffer containing Laemmli (150mM NaCl, 1% Triton X-100, AppliChem; 0.5% sodium deoxycholate Sigma-Aldrich; 50mM Tris-HCl at pH7.3, 2mM EDTA, both Merk; 0.8% protease/phosphatase inhibitor, Cell Signalling) on a concentration of 100µL·10^6cells^{-1} and boiled for 5 minutes. SDS-PAGE of the lysates was performed on 10% or 12% self-casted acrylamide gels. Protein was transferred into PVDF membranes that were incubated with the corresponding antibody. The following antibodies were used: sheep anti-G-CSFR (R&D Systems), rabbit anti-JAK2 (Cell Signalling), rabbit anti-Phospho-JAK2 (Tyr1007/1008, Cell Signalling), mouse anti-STAT3 (Cell Signalling), rabbit anti-Phospho-STAT3 (Tyr705, Cell Signalling), and mouse anti-GAPDH (Merk). Western Blot membranes were scanned with an Odyssey scanner (LI-COR) and analysed with Image Studio Lite software (LI-COR).

Supplemental References

1. Kurz ARM, Pruenster M, Rohwedder I, et al. MST1-dependent vesicle trafficking regulates neutrophil transmigration through the vascular basement membrane. *J. Clin. Invest.* (2016) 126(11):4125–4139. doi: 10.1172/JCI87043.
2. Evrard M, Kwok IWH, Chong SZ, et al. Developmental Analysis of Bone Marrow Neutrophils Reveals Populations Specialized in Expansion, Trafficking, and Effector Functions. *Immunity.* (2018) 48(2):364-79. doi: 10.1016/j.immuni.2018.02.002.

Supplemental Figure 1

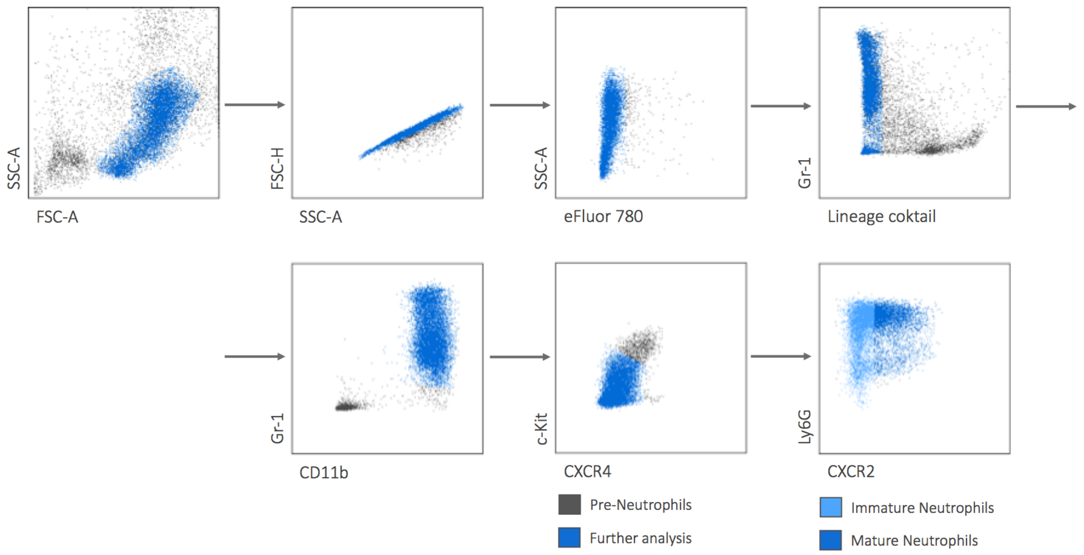

Supplemental Figure 1. Representative plots of neutrophil subsets in the bone marrow panel. Blue highlight indicates the populations selected for the following step in the gating strategy. For the last plots the populations of interest are highlighted as indicated in the legend.

Supplemental Table 1

Antibody	Conjugate	Host	Isotype	Clone	Company
CD3e	PE	Hamster	IgG	145-2C11	BioLegend
CD11b (Mac-1)	PE	Rat	IgG2b k	M1/70	BioLegend
CD11b (Mac-1)	PE-Cy7	Rat	IgG2b k	M1/70	BioLegend
CD11b (Mac-1)	AF 700	Rat	IgG2b k	M1/70	BioLegend
CD45	BV 570	Rat	IgG2b k	IM7	BioLegend
CD45	PerCP-Cy5.5	Rat	IgG2b k	30-F11	BioLegend
CD45	FITC	Rat	IgG2b k	30-F11	BioLegend
CD45	APC	Rat	IgG2b k	30-F11	BioLegend
CD45R (B220)	PE	Rat	IgG2a k	B220	BioLegend
CD90.2 (Thy-1.2)	PE	Rat	IgG2b k	30-H12	BioLegend
CD114 (G-CSFR)	AF 647	Rat	IgG2a	723806	R&D
CD115 (M-CSFR)	PE	Rat	IgG2a k	AFS98	BioLegend
CD115 (M-CSFR)	APC	Rat	IgG2a k	AFS98	BioLegend
CD117 (c-Kit)	BV 421	Rat	IgG2a k	2B8	BioLegend
CD170 (SiglecF)	PE	Rat	IgG2a	E50-2440	BD Pharm
CD182 (CXCR2)	APC	Rat	IgG2a k	SA044G4	BioLegend
CD184 (CXCR4)	FITC	Rat	IgG2b	2B11	Pharmingen
IgG1 k	PE	Mouse	Isotype	P3.6.2.8.1.	Invitrogen
IgG2a k	APC	Rat	Isotype	RTK2758	Biolegend
IgG2a k	AF 647	Rat	Isotype	RTK2758	Biolegend
IgG2b k	FITC	Rat	Isotype	RTK4530	Biolegend
Nk1.1	PE	Mouse	IgG2a k	PK136	BioLegend
Ly6A/E (Sca-1)	PE	Rat	IgG2a	D7	eBioscience
Ly6G/C (Gr-1)	PB	Rat	IgG2b	RB6-8C5	BioLegend
Ly6G/C (Gr-1)	PE-Cy7	Rat	IgG2b k	RB6-8C5	BioLegend
Ly6G	PB	Rat	IgG2a k	1A8	BioLegend
Ly6G	AF 488	Rat	IgG2a k	1A8	BioLegend
Ly6G	PE-Dazzle 594	Rat	IgG2a k	1A8	BioLegend

Supplemental Table 1. List of antibodies for flow cytometry.

Video Legend

Video 1. Time-lapse intravital multi-photon microscopy video of the skull bone marrow of an unstimulated *Lyz2*^{eEGP} mouse showing neutrophils (green) and vessels (red). Projection of 12 z-stacks. Total imaging time of 10 minutes. Scale bar 50µm.

Video 2. Time-lapse intravital multi-photon microscopy video of the skull bone marrow of a *Lyz2*^{eEGP} mouse 2 hours after G-CSF stimulation showing neutrophils (green) and vessels (red). Projection of 12 z-stacks. Total imaging time of 30 minutes. Scale bar 50µm.

Video 3. Time-lapse intravital multi-photon microscopy video of the skull bone marrow of a *Mst1*$^{-/-}$*Lyz2*^{eEGP} mouse 2 hours after G-CSF stimulation showing neutrophils (green) and vessels (red). Projection of 12 z-stacks. Total imaging time of 30 minutes. Scale bar 50µm.
